# Trametinib modulates cancer multidrug resistance by targeting ABCB1 transporter

**DOI:** 10.18632/oncotarget.3820

**Published:** 2015-04-14

**Authors:** Jian-Ge Qiu, Yao-Jun Zhang, Yong Li, Jin-Ming Zhao, Wen-Ji Zhang, Qi-Wei Jiang, Xiao-Long Mei, You-Qiu Xue, Wu-Ming Qin, Yang Yang, Di-Wei Zheng, Yao Chen, Meng-Ning Wei, Zhi Shi

**Affiliations:** ^1^ Department of Cell Biology and Institute of Biomedicine, College of Life Science and Technology, Jinan University, National Engineering Research Center of Genetic Medicine, Guangdong Provincial Key Laboratory of Bioengineering Medicine, Guangzhou, Guangdong, China; ^2^ Department of Hepatobiliary Surgery, Cancer Center, Sun Yat-Sen University, Guangzhou, Guangdong, China; ^3^ Department of Gastrointertinal Surgery and General Surgery, Guangdong General Hospital, Guangdong Academy of Medical Sciences, Guangzhou, Guangdong, China; ^4^ Department of Thoracic Surgery, The Sixth Affiliated Hospital, Sun Yat-sen University, Guangzhou, Guangdong, China

**Keywords:** trametinib, multidrug resistance, ABCB1, chemotherapy

## Abstract

Overexpression of adenine triphosphate (ATP)-binding cassette (ABC) transporters is one of the main reasons of multidrug resistance (MDR) in cancer cells. Trametinib, a novel specific small-molecule mitogen-activated extracellular signal-regulated kinase (MEK) inhibitor, is currently used for the treatment of melanoma in clinic. In this study, we investigated the effect of trametinib on MDR mediated by ABC transporters. Trametinib significantly potentiated the effects of two ABCB1 substrates vincristine and doxorubicin on inhibition of growth, arrest of cell cycle and induction of apoptosis in cancer cells overexpressed ABCB1, but not ABCC1 and ABCG2. Furthermore, trametinib did not alter the sensitivity of non-ABCB1 substrate cisplatin. Mechanistically, trametinib potently blocked the drug-efflux activity of ABCB1 to increase the intracellular accumulation of rhodamine 123 and doxorubicin and stimulates the ATPase of ABCB1 without alteration of the expression of ABCB1. Importantly, trametinib remarkably enhanced the effect of vincristine against the xenografts of ABCB1-overexpressing cancer cells in nude mice. The predicted binding mode showed the hydrophobic interactions of trametinib within the large drug binding cavity of ABCB1. Consequently, our findings may have important implications for use of trametinib in combination therapy for cancer treatment.

## INTRODUCTION

Cancer cells with multidrug resistance (MDR) to chemotherapeutic drugs significantly reduces the efficacy of cancer chemotherapy [1]. Various mechanisms are involved in the MDR of cancer, including induction of the antiapoptotic machinery, increase of the intracellular drug efflux, reduce of the drug uptake, and so on [2]. Overexpressing the adenine triphosphate (ATP)-binding cassette (ABC) transporters, particularly ABCB1 (MDR1/P-glycoprotein), ABCC1 (MRP1) and ABCG2 (BCRP), are one of most common reasons to result in MDR in cancer cells [3-6]. For instance, ABCB1 can transport multiple types of chemotherapeutic drugs out of cells, such as the taxanes (paclitaxel, docetaxel), epipodophyllotoxins (etoposide), vinca alkaloids (vincristine, vinblastine), and anthracyclines (doxorubicin, epirubicin), and this process is coupled to the energy of ATP hydrolysis on the ATPase domain [7-10]. Therefore, inhibition of these transporters will restore the sensitivity of MDR cancer cells to chemotherapeutic agents, and may permit a successful chemotherapy to patients with MDR cancer [11, 12].

Trametinib (Mekinist, GSK1120212) is a novel selective and highly potent small molecular inhibitor of mitogen-activated extracellular signal-regulated kinase (MEK) with a half maximum IC_50_ of 0.7-14.9 nM for MEK1 and MEK2, which are the central hubs of mitogen-activated protein kinase (MAPK) pathway to control oncogenic cell proliferation, survival, invasion, angiogenesis and death, etc [13-15]. The preclinical experiments demonstrated trametinib had broad anticancer activity in multiple cancer models by inducing cell cycle arrest, apoptosis and growth inhibition *in vitro* and *in vivo*, especially in cancer cells with activating mutations of BRAF and KRAS in the MAPK pathway [14]. In a multicentre phase 1 dose-escalation trial of 206 patients with advanced solid tumors to assesse the safety, pharmacokinetics, pharmacodynamics, and efficacy data of trametinib, the maximum tolerated dose was 3 mg once daily, the effective half-life was around 4 days, and active pathway inhibition and clinical activity were recorded [16]. Another phase 1 study in 97 patients with advanced melanoma showed substantial clinical activity of trametinib in melanoma [17]. The following open-label, two-stage, phase II trial with two cohorts in patients with metastatic BRAF-mutant melanoma observed significant clinical activity of trametinib with the most common side effects including skin-related toxicity, peripheral edema, nausea, pruritis, diarrhea, and fatigue [18]. In the phase 3 trial in 322 patients who had metastatic melanoma with V600E or V600K BRAF mutation, trametinib improved progression-free survival and overall survival times in comparesion with dacarbazine or paclitaxel [19]. Based on these data, trametinib was approved by FDA as monotherapy for use in patients with V600E or V600K BRAF mutated unresectable or metastatic melanoma in May 2013. Moreover, FDA granted accelerated approval to the combination therapy of trametinib with a BRAF inhibitor dabrafenib in the aforementioned patients in January 2014, based on the improved median progression-free survival and response rate shown with combination therapy [20]. Currently, investigation of trametinib in combination with other chemotherapeutical agents for the treatment of multiple types of cancers is ongoing. In this study, we demonstrate that trametinib significantly sensitizes ABCB1-medidated MDR cancer cells to chemotherapeutic agents *in vitro* and *in vivo* by directly antagonizing the drug-efflux activity of ABCB1.

## RESULTS

### Trametinib enhances the sensitivity of ABCB1-substrate chemotherapeutic agents in the ABCB1-overexpressing cells

**Figure 1:**
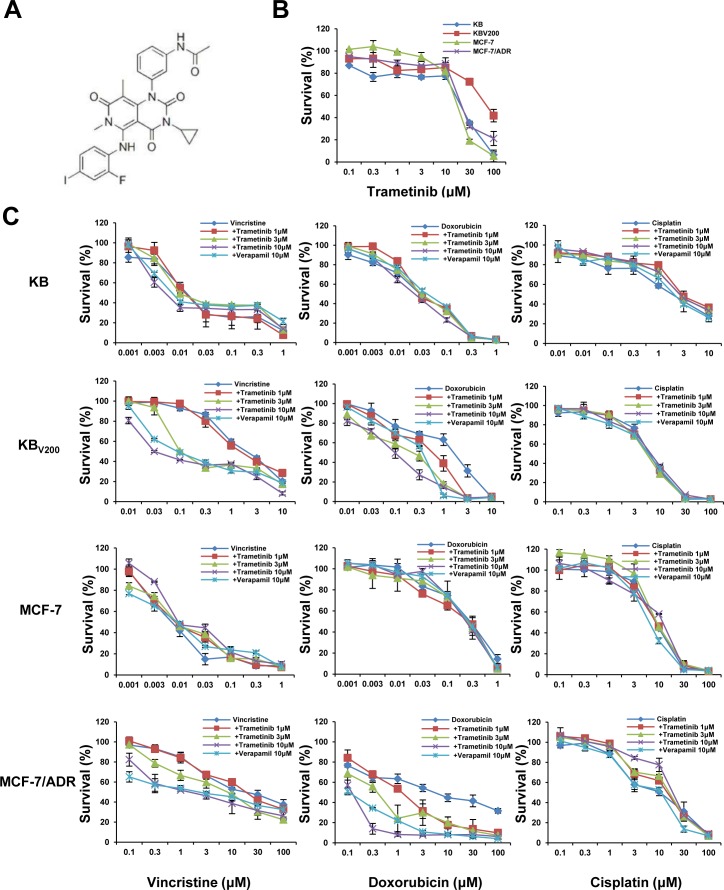
Trametinib enhances the sensitivity of ABCB1-substrate chemotherapeutic agents in the ABCB1-overexpressing cells Cells were treated with the indicated concentrations of trametinib (**A**) or other agents for 72 h, and cell survival was measured by MTT assay. The representative growth curve of KB, KB_V200_, MCF-7 and MCF-7/ADR cells treated with trametinib alone (**B**) or in combination with vincristine, doxorubicin and cisplatin (**C**) are shown.

To investigate the effects of trametinib on ABCB1-mediated MDR in cancer cells, we firstly examined the cytotoxicity of trametinib in two ABCB1-overexpressing cells KB_V200_ and MCF-7/ADR and their parental cells KB and MCF-7 by MTT assay. As shown in Figure 1B, over 80% of all four cells were viable after treated with trametinib at 10 μM, indicating that this dose could be used as the highest concentration to explore the ability of trametinib on enhancing the sensitivity of chemotherapeutic drugs in ABCB1-overexpressing MDR cancer cells. We then tested the cytotoxicity of combination of trametinib with two ABCB1 substrates vincristine and doxorubicin and one non-ABCB1 substrate cisplatin at the various concentrations. The summary IC_50_ values and survival curves were shown in Table 1 and Figure 1C. Compared with KB and MCF-7 cells, KB_V200_ and MCF-7/ADR cells exhibited high resistance to vincristine and doxorubicin but not to cisplatin. Trametinib dose-dependently decreased the IC_50_ values of vincristine and doxorubicin in both KB_V200_ and MCF-7/ADR cells but not in KB and MCF-7 cells, which was similar to the effects of the known ABCB1 inhibitor verapamil. Furthermore, trametinib did not significantly alter the cytotoxicity of cisplatin in either MDR or parental cells. In addition, we also detected the effects of trametinib on ABCC1 and ABCG2-mediated MDR, and found that trametinib at 10 μM did not reduce the resistances of vincristine (also the substrate of ABCC1) in ABCC1-overexpressing cells KB-CV60 and doxorubicin (also the substrate of ABCG2) in ABCG2-overexpressing cells S1-M1-80 (Supplementary Figure 1). Together, our results demonstrated that trametinib significantly enhanced the sensitivity of ABCB1-substrate chemotherapeutic agents in the ABCB1-overexpressing cells, suggesting trametinib is able to antagonize ABCB1-mediated cancer MDR *in vitro*.

**Table 1:** Summary of the effects of trametinib on enhancing the sensitivity of vincristine, doxorubicin and cisplatin in cancer cells

Compounds (μM)	IC_50_±SD (fold-reversal)			
	KB	KB_v200_	MCF-7	MCF-7/ADR
Vincristine	0.020±0.007(1.000)	2.044±0.193(1.000)	0.012±0.006(1.000)	19.474±1.183(1.000)
+Trametinib 1μM	0.015±0.002(1.333)	1.745±0.087(1.171)	0.014±0.007(0.857)	20.080±0.974(0.970)
+Trametinib 3μM	0.014±0.006(1.429)	0.166±0.093(12.313)*	0.013±0.006(0.923)	8.960±0.294(2.173)*
+Trametinib 10μM	0.012±0.009(1.667)	0.042±0.018(48.667)**	0.012±0.003(1.000)	2.209±0.774(8.816)*
+Verapamil 10μM	0.009±0.001(2.222)	0.083±0.015(24.627)*	0.013±0.005(0.923)	2.102±0.437(9.265)*
Doxorubicin	0.034±0.008(1.000)	1.662±0.235(1.000)	0.254±0.037(1.000)	4.749±1.957(1.000)
+Trametinib 1μM	0.031±0.004(1.097)	0.386±0.413(4.306)*	0.231±0.055(1.100)	1.113±0.086(4.267)*
+Trametinib 3μM)	0.028±0.002(1.214)	0.250±0.012(6.648)*	0.199±0.081(1.276)	0.517±0.181(9.186)*
+Trametinib 10μM	0.024±0.001(1.417)	0.096±0.003(17.313)*	0.185±0.090(1.373)	0.234±0.143(20.295)*
+Verapamil 10μM	0.046±0.003(0.739)	0.232±0.226(7.164)*	0.260±0.013(0.977)	0.145±0.047(32.752)*
Cisplatin	2.357±0.402(1.000)	6.838±0.403(1.000)	7.992±1.588(1.000)	11.229±0.670 (1.000)
+Trametinib 1μM	2.863±0.021(0.823)	6.221±0.823(1. 099)	8.789±0.692(0.909)	21.168±6.503(0.530)
+Trametinib 3μM	2.390±0.367(0.986)	5.481±1.174(1.248)	9.013±0.541(0.887)	23.884±8.149(0.470)
+Trametinib 10μM	2.34±0.4789(1.007)	6.317±1.634(1.082)	12.064±1.495(0.660)	22.664±2.426(0.0.495)
+Verapamil 10μM	2.459±0.329(0.959)	6.606±0.321(1.035)	7.560±0.365(1.057)	11.711±0.397(0.959)

The fold-reversal value was calculated by dividing the IC_50_ for cells with the anticancer in the absence of trametinib or verapamil by that obtained in the presence of trametinib or verapamil.

* *P*<0.05 and

** *P*<0.01 vs. corresponding control (n=3).

### Trametinib in combination with ABCB1-substrate chemotherapeutic agents induces cell cycle arrest in the ABCB1-overexpressing cells

**Figure 2:**
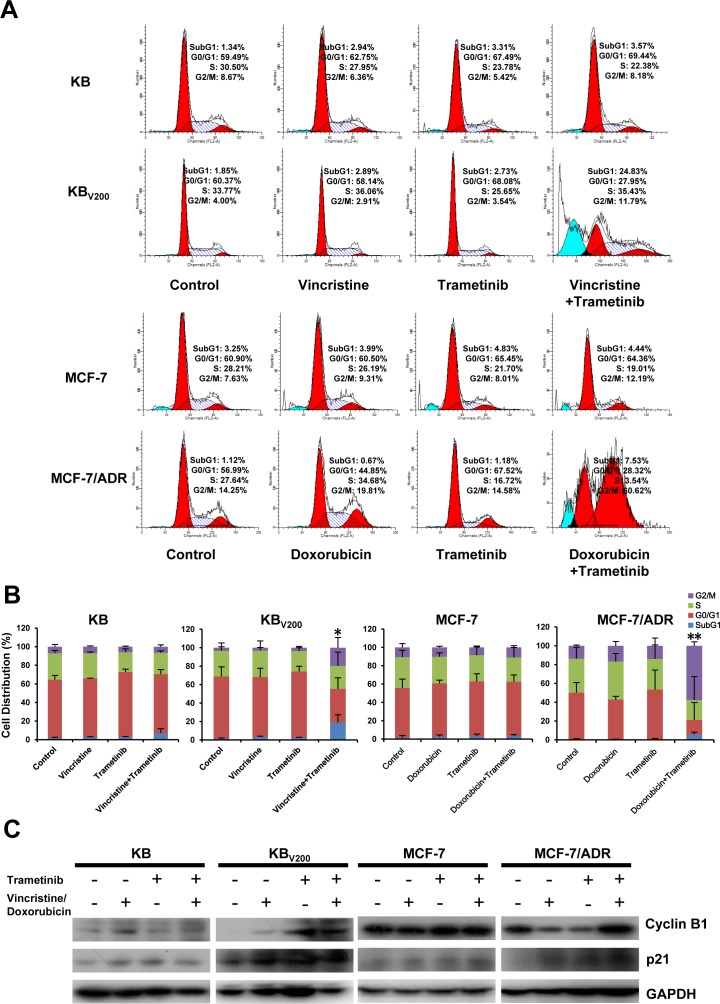
Trametinib in combination with ABCB1-substrate chemotherapeutic agents induces cell cycle arrest in the ABCB1-overexpressing cells Cells were treated with the indicated agents for 48 hours, and the distribution of cell cycle was detected by FCM with PI staining. The protein expression was examined by Western blot after lysing cells, and GAPDH was used as loading control. The concentrations of each agent were used as follow: vincristine 0.03 μM in KB and 0.3 μM in KB_V200_, doxorubicin 0.01 μM in MCF-7 and 1 μM in MCF-7/ADR, trametinib 10 μM in all four cells. The representative charts (**A**), quantified data (**B**) and Western blot results (**C**) are shown. *P < 0.05 and **P < 0.01 vs. corresponding control (*n* = 3).

To evaluate the effects of trametinib in combination with chemotherapeutic agents in the ABCB1-overexpressing cells, cell cycle distribution and the related proteins were detected by FCM and Western blot, respectively. As shown in Figure 2A and 2B, co-treatment with trametinib and vincristine significantly increased the cell population of sub-G1 and G2/M phase and the levels of G2/M-phase dominant proteins cyclin B1 and cyclin-dependent kinase inhibitor p21 in comparison with trametinib or vincristine alone treatment in KB_V200_ cells but not in KB cells. Similarly, co-treatment with trametinib and doxorubicin significantly increased the cell population of sub-G1 and G2/M phase and the protein levels of cyclin B1 and p21 in comparison with trametinib or doxorubicin alone treatment in MCF-7/ADR cells but not in MCF-7 cells.

### Trametinib in combination with ABCB1-substrate chemotherapeutic agents induces apoptosis in the ABCB1-overexpressing cells

**Figure 3:**
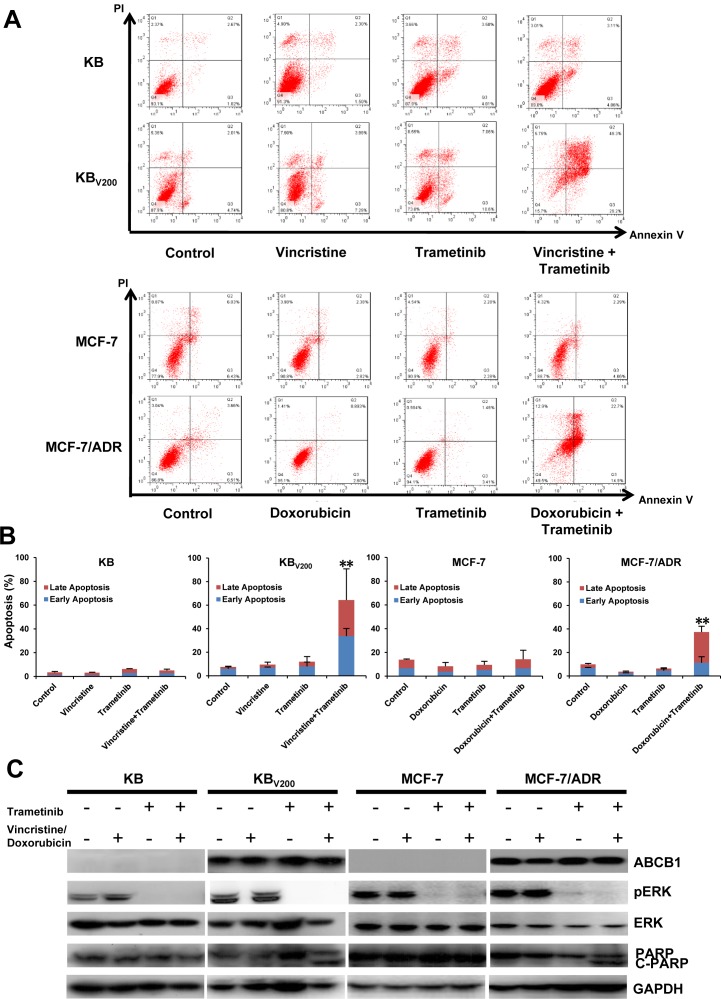
Trametinib in combination with ABCB1-substrate chemotherapeutic agents induces apoptosis in the ABCB1-overexpressing cells Cells were treated with the indicated agents for 48 hours, and the apoptosis was detected by FCM Annexin V/PI staining. The proportions of Annexin V+/PI- and Annexin V+/PI+ cells indicated the early and late stage of apoptosis. The protein expression was examined by Western blot after lysing cells, and GAPDH was used as loading control. The concentrations of each agent were used as follow: vincristine 0.03 μM in KB and 0.3 μM in KB_V200_, doxorubicin 0.01 μM in MCF-7 and 1 μM in MCF-7/ADR, trametinib 10 μM in all four cells. The representative charts (**A**), quantified data (**B**) and Western blot results (**C**) are shown. *P < 0.05 and **P < 0.01 *vs.* corresponding control (*n* = 3).

To further estimate the effects of trametinib in combination with chemotherapeutic agents in the ABCB1-overexpressing cells, cell apoptosis and the related proteins were also detected by FCM and Western blot, respectively. As shown in Figure 3A and 3B, co-treatment with trametinib and vincristine dramatically enhanced the early apoptosis (Annexin V+/PI-) and late apoptosis (Annexin V+/PI+) and the protein levels of apoptotic marker cleaved PARP (C-PARP) in comparison with trametinib or vincristine alone treatment in KB_V200_ cells but not in KB cells. Similarly, co-treatment with trametinib and doxorubicin dramatically enhanced the apoptosis and the protein levels of C-PARP in comparison with trametinib or doxorubicin alone treatment in MCF-7/ADR cells but not in MCF-7 cells. In addition, the protein levels of phosphorylated ERK (pERK) were completely blocked by trametinib in all four cells.

### Trametinib in combination with vincristine inhibits the growth of KB_V200_ xenografts in nude mice

**Figure 4:**
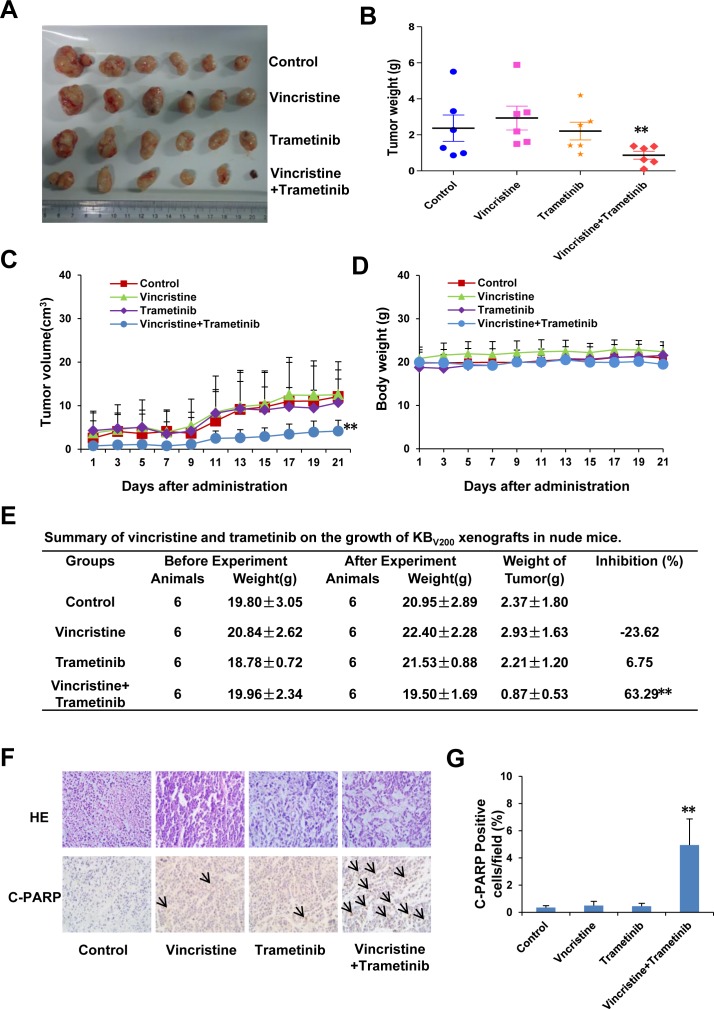
Trametinib in combination with vincristine inhibits the growth of KB_**V200**_ xenografts in nude mice Each mouse was injected subcutaneously with KB_V200_ cells (2 × 10^6^ in 100 μl of medium) under the shoulder. When the subcutaneous tumors were approximately 0.3 × 0.3 cm^2^ (two perpendicular diameters) in size, mice were randomized into four groups and treated with the following regimens: vehicle alone (0.9% saline), vincristine (0.1 mg/kg, intraperitoneally), trametinib (3 mg/kg, orally), and the combination of vincristine with trametinib every two days. The body weights of mice and tumor volume were recorded. The mice were anaesthetized after experiment, and tumor tissue was excised from the mice and weighted. The original tumors (**A**), tumor weight (**B**), tumor volume (**C**), body weight (**D**) and summary data (**E**) are shown. The representative H&E and immunohistochemical C-PARP staining of KB_V200_ xenografts (**F**), and quantified C-PARP positive cells (**G**) are also presented. The values presented are the means ± SD for each group. *P < 0.05 and **P < 0.01 *vs.* corresponding control (*n* = 6).

To confirm the ability of trametinib antagonizing ABCB1-mediated cancer MDR *in vivo*, KB_V200_ xenografts models were generated in the nude mice. As shown in Figure 4A-4C, treatment with trametinib or vincristine alone did not inhibit the growth of KB_V200_ xenografts, but co-treatment with trametinib and vincristine obviously inhibited the growth of KB_V200_ xenografts with the inhibition ratio of 63.29% (Figure 4E). Furthermore, there was no loss of mice body weight in the combination group, suggesting that the combination regimen at the indicated dose did not cause toxicity in mice (Figure 4D). Additionally, the results of immunohistochemical staining showed that the percentage of C-PARP positive cells in KB_V200_ xenografts was significantly increased in the combination group in comparison with vincristine or trametinib alone group (Figure 4F).

### Trametinib increases the intercellular accumulation of rhodamine 123 and doxorubicin in ABCB1-overexpressing cells

**Figure 5:**
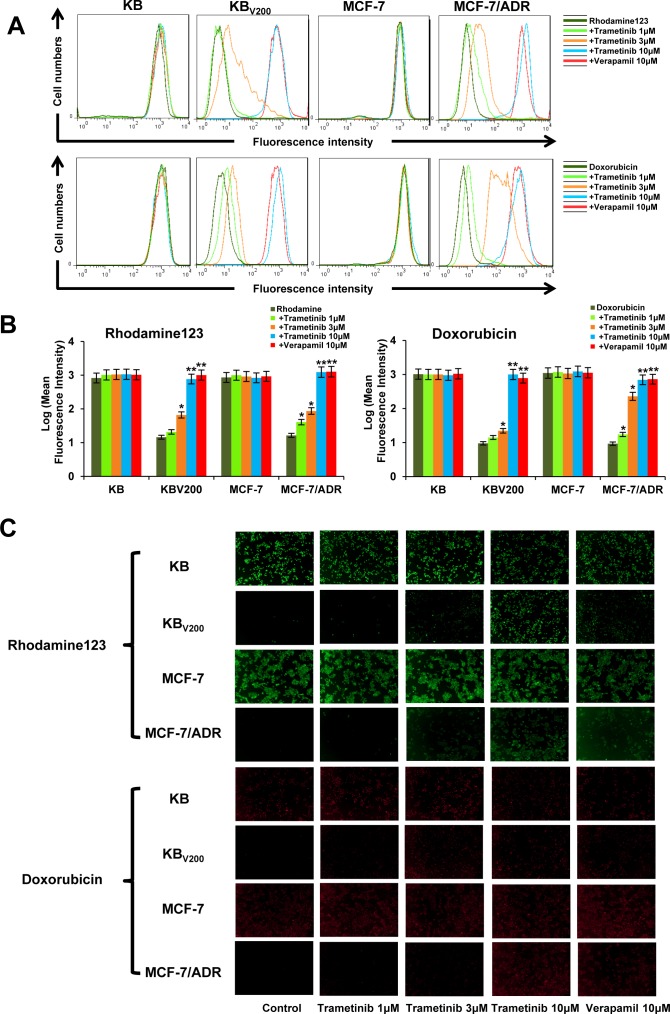
Trametinib increases the intercellular accumulation of rhodamine 123 and doxorubicin in ABCB1-overexpressing cells Cells were incubated with 10μM rhodamin 123 or doxorubicin for another 2 hours at 37°C after pre-treated with the indicated concentrations of trametinib and verapemail for 1 hour at 37 °C, measured by FCM and photographed by fluorescent microscope. The representative charts (**A**), quantified data (**B**) and graphs (**C**) are shown. *P < 0.05 and **P < 0.01 *vs.* corresponding control (*n* = 3).

To examine whether trametinib antagonizing ABCB1-mediated cancer MDR is owing to inhibition of the transporter activity of ABCB1, we measured the intracellular levels of two ABCB1 substrates rhodamine 123 and doxorubicin in the presence or absence of trametinib. As shown in Figure 5A and 5B, the intracellular levels of both rhodamine 123 and doxorubicin in KB_V200_ and MCF-7/ADR cells were significantly lower than those in KB and MCF-7 cells, respectively. Trametinib dose-dependently increased the intracellular levels of rhodamine 123 and doxorubicin in both KB_V200_ and MCF-7/ADR cells but not in KB and MCF-7 cells, which was equal to the effects of verapamil, suggesting that trametinib is able to directly inhibiting the drug efflux function of ABCB1.

### Trametinib stimulates the ATPase activity of ABCB1 and does not alert the expression of ABCB1

**Figure 6:**
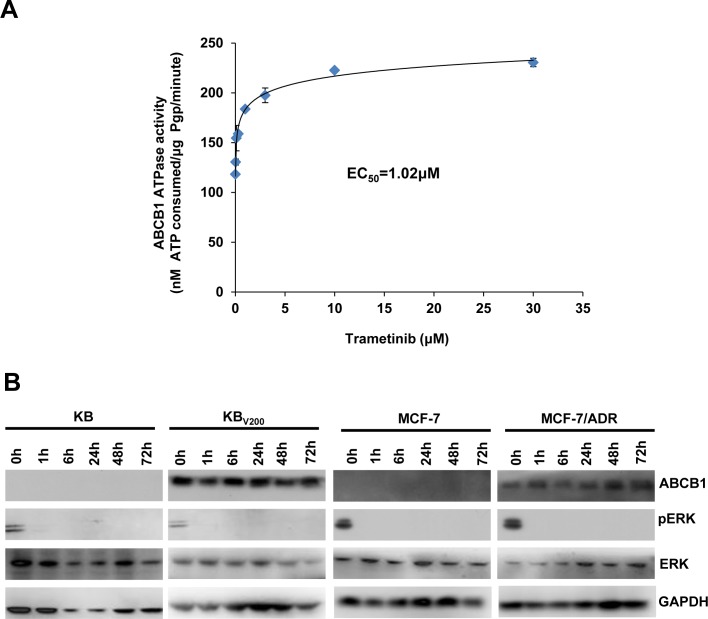
Trametinib stimulates the ATPase activity of ABCB1 and does not alert the expression of ABCB1 The Vi-sensitive ATPase activity of ABCB1 (**A**) in membrane vesicles was determined with different concentration (0.01, 0.03, 0.1, 0.3, 1, 3, 10 and 30 μM) of trametinib. Cells were treated with trametinib at 10 μM for the indicated time points, and the protein expression was examined by Western blot after lysing cells, and GAPDH was used as loading control. The representative Western blot results (**B**) were shown.

The transporter function of ABCB1 is coupled to ATP hydrolysis, which is stimulated in the presence of ABCB1 substrates. To assess the effects of trametinib on the ATPase activity of ABCB1, we detected the ABCB1-mediated ATP hydrolysis with various concentrations of trametinib. As shown in Figure 6A, trametinib enhanced the ATPase activity of ABCB1 in the dose-dependent manner with the EC_50_ value of 1.02 μM, suggesting that trametinib is the substrate of ABCB1. In addition, the reversal of ABCB1-mediated MDR can be achieved either by inhibiting its pump activity or by decreasing its expression. To study the effect of trametinib on ABCB1 expression, the protein levels were detected by Western blot after treatment with trametinib at 10 μM at the various time points. The results showed that the protein levels of pERK were totally inhibited after trametinib treatment as soon as for 1h in all four cells, but the protein levels of ABCB1 were not altered in KB_V200_ and MCF-7/ADR cells after trametinib treatment even up to 72 hours (Figure 6B). These data indicate that trametinib is able to inhibit the activity of MEK but unable to alter the protein expression of ABCB1.

### Model for binding of trametinib to ABCB1

**Figure 7:**
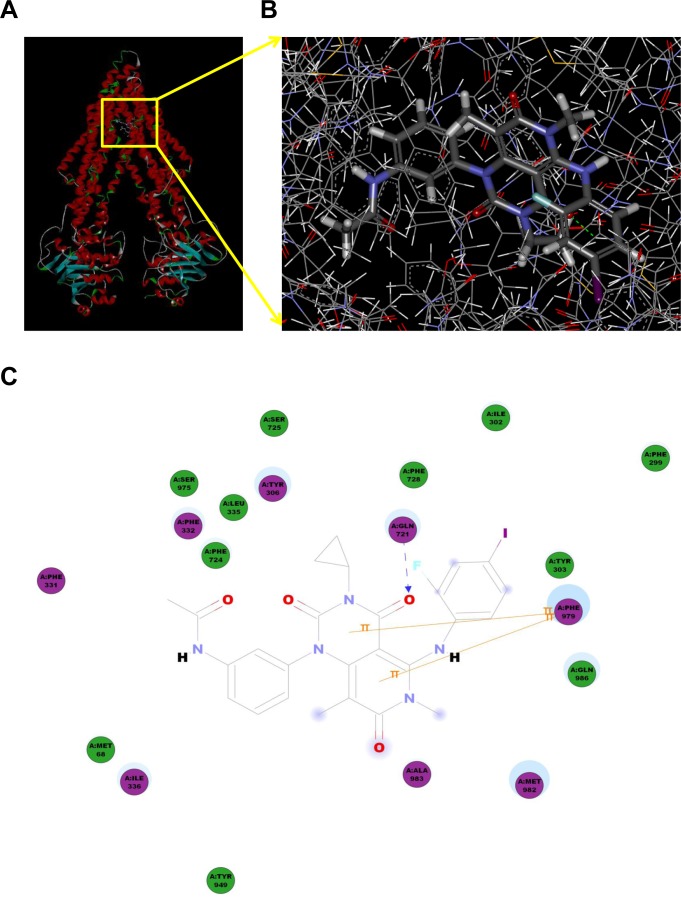
Model for binding of trametinib to ABCB1 The ribbon diagram of 3D structure conformation (**A**) and the optimal predicted binding mode (**B**) of trametinib within mouse ABCB1 binding site are shown. Important amino acids are depicted as lines with the atoms colored (carbon, gray; hydrogen, white; nitrogen, blue and oxygen, red), and trametinib is shown as ball and stick model with the atoms colored (carbon, gray; hydrogen, white; nitrogen, blue; oxygen, red; iodine; violet and fluorine; sky-blue). Dotted green line indicates hydrogen bonding interaction. The 2D plane diagram of trametinib-ABCB1 interaction is also presented. Dotted blue line represents the interaction site of trametinib and ABCB1.

To understand the mechanism of binding of trametinib to ABCB1 at a molecular level, docking studies were performed with the crystal structure of mouse Mdr3 as represented by ABCB1-QZ59-RRR, ABCB1-QZ59-SSS and ABCB1-verapamil. As shown in Figure 7A and 7B, the predicted binding mode showed the hydrophobic interactions of trametinib within the large drug binding cavity of ABCB1. Trametinib was stabilized through specific interactions such as hydrogen bonding and nonspecific interactions such as hydrophobic interactions with residues in the drug-binding pocket of ABCB1. The hydrogen bond acceptor oxygen atom at the position of the carbanyl group showed hydrogen bonding interaction with the side chain of Gln721 (CO-Gln721). The pyrimidine ring and pyridine ring of trametinib interacted with Phe979 through π−π stacking. The other groups of trametinib might be mainly stabilized through hydrophobic contacts within the large hydrophobic pocket formed by the side chains of Met68, Phe299, Ile302, Tyr303, Tyr306, Phe337, Leu335, Ile336, Phe724, Ala725, Phe728, Tyr949, Ser975, Met982, Ala983 and Gln986 (Figure 7C and Supplementary Table 1).

## DISCUSSION

Targeted therapy currently is one of the major strategies for cancer therapy, which suppresses the growth of cancer cells by inhibiting specific molecular targets required for cancer growth. Small molecular agent is the main category of targeted therapy. Until now, a number of small molecules for cancer targeted therapy were successfully used in clinic, such as BCL-Abl inhibitors imatinib and dasatinib for chronic myelogenous leukemia, epidermal growth factor receptor (EGFR) inhibitors gefitinib and erlotinib for non small cell lung cancer, vascular epidermal growth factor receptor (VEGFR) inhibitors sorafenib and sunitinib for renal cell carcinoma, etc. However, emerging literatures suggest that in addition to mutations of targets, overexpression of ABC transporters may contribute to the development of resistance to these small molecular agents [21, 22]. We and others have reported that several small molecular agents, including imatinib [23], dasatinib [24], gefitinib [25], erlotinib [26], icotinib [27], sorafenib [28], sunitinib [29], lapatinib [30], apatinib [31], afatinib [32] and so on, are substrates or modulators of ABCB1 or ABCG2. Therefore, when the combination of these small molecules with ABCB1- or ABCG2-substrate agents is applied in the clinic, it should pay attention to the plasma concentrations and tissue distribution of these agents which may be interfered with each other, resulting in unexpected adverse effects. In addition, it is necessary to investigate the interactions of novel small molecules for cancer targeted therapy with ABC transporters.

In the present study, we showed that trametinib significantly potentiated the effects of vincristine and doxorubicin on inhibition of growth, arrest of cell cycle and induction of apoptosis in cancer cells overexpressed ABCB1, but not ABCC1 and ABCG2. Mechanistically, trametinib potently blocked the drug-efflux activity of ABCB1 to increase the intracellular accumulation of rhodamine 123 and doxorubicin and stimulates the ATPase of ABCB1 without alteration of the expression of ABCB1, suggesting that trametinib may work as the substrate of ABCB1 to inhibit its function. This is consistent with a recent publication, which demonstrated ABCB1 plays an important role in limiting brain distribution of trametinib, indicating that trametinib is the substrate of ABCB1 [33]. Consequently, when the combination of trametinib with ABCB1-substrate agents is adminstrated *in vivo*, the safety and efficacy of these agents may be affected mutually. In our case, the combination of trametinib with vincristine dramatically inhibited the growth of ABCB1-overexpressing xenograft tumors in nude mice without loss of body weight, suggesting that the combination regimen herein is safe and efficient. Additionally, it has been reported that inhibition of the growth of patient-derived pancreatic cancer xenografts with trametinib was augmented by combined treatment with lapatinib [34], or panitumumab and trastuzumabr [35]. Trametinib also enhanced the efficacy of 5-fluorouracil on human colon cancer cells [36], and have synergistic effects with metformin on cell viability and tumor growth in NRAS mutant cancer [37]. In a phase Ib study in 31 patients with advanced solid tumours, administration of trametinib in combination with gemcitabine was feasible, and the addition of trametinib might increase gemcitabine-associated myelosuppression although pharmacokinetics suggested no change in exposures of either drug in combination [38]. However, in the following phase II study in 160 patients with previously untreated metastatic pancreas cancer, the addition of trametinib to gemcitabine did not improve overall survival, progression-free survival, overall response rate and duration of response [39]. Trametinib could safely be given with weekly paclitaxel at the full monotherapy dose with promising progression free and overall survival in the phase I trial in patients with melanoma lacking a V600 BRAF mutation [40]. Concurrent treatment with trametinib and the mTOR inhibitor everolimus resulted in frequent treatment-related adverse events, and pharmacodynamic data did not indicate drug-drug interactions between these two agents in the phase Ib trial in 67 patients with advanced solid tumors [41]. In a phase I study in 20 patients with solid tumors and multiple myeloma, trametinib in combination with the AKT inhibitor afuresertib were poorly tolerated at continuous daily dosing, but tolerable at intermittent dosing schedule [42]. In another phase Ib trial in 113 patients with advanced solid tumors, co-treatment with trametinib and PI3K inhibitor buparlisib showed promising antitumor activity for patients with KRAS-mutant ovarian cancer, but long-term tolerability of the combination was challenging due to frequent dose interruptions and reductions for toxicity [43]. Together, the combination of trametinib with other anticancer agents, especially ABCB1-substrate chemotherapeutic agents, need to be further investigated in the either preclinical models or clinical trials.

To sum up, our results shows that trametinib significantly antagonizes ABCB1-mediated cancer MDR *in vitro* and *in vivo* by directly blocking the drug-efflux function of ABCB1, which is supported by the predicted binding mode that showed the hydrophobic interactions of trametinib within the large drug binding cavity of ABCB1. Consequently, our findings may have important implications for use of trametinib in combination therapy for cancer treatment.

## MATERIALS AND METHODS

### Cell culture and reagents

The ABCB1-overexpressing MDR cancer cells, KB_V200_ and MCF-7/ADR, were generated from human cancer cells KB and MCF-7 by stepwise exposure to increasing doses of vincristine and doxorubicin, respectively [44]. The ABCC1-overexpressing MDR cancer cells KB-CV60 were derived from KB-3-1 cells and maintained in medium with 1 μg/mL of cepharanthine and 60 ng/mL of vincristine [45]. The ABCG2-overexpressing MDR cancer cells S1-M1-80 were established from S1 cells and maintained in the medium with 80 μM of mitoxantrone [46]. All cells were cultured in Dulbecco's modified Eagle's medium (DMEM) supplemented with 10% fetal bovine serum (FBS), penicillin (100 U/ml) and streptomycin (100 ng/ml) in a humidified incubator at 37°C with 5% CO2. Trametinib was obtained from ApeBio. Vincristine, doxorubicin and cisplatin were ordered from LC Laboratories. Verapamil and rhodamine 123 were purchased from Sigma-Aldrich. Methylthiazolyldiphenyl-tetrazolium bromide (MTT), propidium iodide (PI), hematoxylin and other chemicals were purchased from Sangon Biotech (Shanghai). Pgp-Glo^TM^ Assay Systems (V3601) was acquired from Promega. Anti-pErk (4370), Anti-Erk (9102), Anti-PARP (9542), Anti-cleaved PARP (5625) and Anti-XIAP (2045) antibodies were from Cell Signaling Technologies. Anti-cyclin B (61029) and Anti-p21 (554262) antibodies were from BD Biosciences. Anti-ABCB1 (SC-13131) antibody was from Santa Cruz Biotechnology. Anti-GAPDH (KM9002) antibody was from Tianjin Sungene Biotech.

### Cell viability assay

Cells were seeded into a 96-well plate at a density of 5 × 10^3^ cells/well and treated with various concentrations of agents for 72 hours. MTT was added to each well at a final concentration of 0.5 mg/ml. After incubation for 4 hours, formazan crystals were dissolved in 100 ml of DMSO, and absorbance at 570 nm was measured by plate reader. The concentrations required to inhibit growth by 50% (IC_50_) were calculated from survival curves as previously described [47].

### Cell cycle assay

Cells were harvested and washed twice with cold phosphate-buffered saline (PBS), then fixed with ice-cold 70% ethanol for 30 minutes at 4 °C. After centrifugation at 200 × g for 10 minutes, cells were washed twice with PBS and resuspended with 0.5 ml PBS containing PI (50 μg/ml), 0.1% Triton X-100, 0.1% sodium citrate, and DNase-free RNase (100 μg/ml), and detected by FACSCalibur flow cytometer (FCM) after 15 minutes incubation at room temperature in the dark. Fluorescence was measured at an excitation wavelength of 480 nm through a FL-2filter (585 nm). Data were analyzed using ModFit LT 3.0 software.

### Apoptosis assay

Cells were harvested and washed twice with PBS, stained with Annexin V-FITC and propidium iodide (PI) in the binding buffer, and detected by FCM after 15 minutes incubation at room temperature in the dark. Fluorescence was measured at an excitation wave length of 480 nm through FL-1 (530 nm) and FL-2 filters (585 nm). The early apoptotic cells (Annexin V positive only) and late apoptotic cells (Annexin V and PI positive) were quantified with the FlowJO software as previously described [48].

### Western blot analysis

Cells were harvested and lysed in RIPA buffer (1% NP-40, 0.5% sodium deoxycholate, 0.1% SDS, 10 ng/ml PMSF, 0.03% aprotinin, 1μM sodium orthovanadate) at 4°C for 30 minutes. After centrifuged for 10 minutes at 14,000 × g, supernatants were collected. Protein concentration was quantified using with Bradford assay. Proteins were separated on 12% SDS-PAGE gels and transferred to polyvinylidene difluoride membranes. Membranes were blocked with 5% BSA and incubated with the indicated primary antibodies. Corresponding horseradish peroxidase-conjugated secondary antibodies were used against each primary antibody. Proteins were detected using the chemiluminescent detection reagents and films.

### Nude mice xenograft tumor assay

Balb/c nude mice were obtained from the Guangdong Medical Laboratory Animal Center and maintained with sterilized food and water. Six female nude mice with 5 weeks old and 18-22 g weight were used for each group. Every mouse was injected subcutaneously of the KB_V200_ cells (3×10^6^ in 100μl of DMEM) under the right shoulder. When the subcutaneous tumors were approximately 0.5 × 0.5 cm^2^ (two perpendicular diameters) in size, the mice were randomized into four groups and treated with the following regimens: vehicle alone (0.9% saline), vincristine (0.1 mg/kg, intraperitoneally), trametinib (3 mg/kg, orally), and the combination of vincristine with trametinib every two days. The body weights of the animals and the two perpendicular diameters (A and B) were recorded every 2 days. The tumor volume (V) was calculated as:

$$\text{v}=\frac{\text{π}}{6}{\left(\frac{\text{A+B}}{2}\right)}^{3}$$

The mice were anaesthetized after experiment, and tumor tissue was excised from the mice and weighted. The rate of inhibition (IR) was calculated according to the formula:

$$\text{IR}=\text{1}-\frac{\text{Mean tumor weight of experimental group}}{\text{Mean tumor weight of control group}}\times 100\%$$

### Immunohistochemistry assay

Immunohistochemistry assay was performed with a microwave-enhanced avidin-biotin stainingmethod as previously described [49, 50]. Formalin-fixed, paraffin-embedded tumor tissue slides were deparaffinized using xylene and graded ethyl alcohol and then rinsed in water. Antigen retrieval was performed by boiling the slides in 0.01 M citrate buffer in a microwave oven for 10 minutes and cooling at room temperature. The slides were then incubated with 0.05% Triton-X100 in PBS for 5 minutes, followed by sequential treatment in a humidified chamber after quenching endogenous peroxides with 3% H_2_O_2_ in MeOH: blocking serum with avidin for 20 minutes, anti-cleaved-PARP antibody overnight at 4 °C, secondary antibody for 20 minutes, hydrogen peroxidase for 15 minutes, and peroxidase substrate solution for 20 minutes at room temperature. The stained slides were then counterstained with hematoxylin and coverslipped. The percentages of C-PARP positive cells were quantified as the average of five fields for each slide.

### Rhodamine 123 and doxorubicin accumulation assay

Cells were seeded into a 6-well plate at a density of 2.5 × 10^5^ cells/well, pre-incubated with or without inhibitors for 1 hour at 37 °C, and incubated with 10 μM rhodamine 123 or doxorubicin for another 2 hours at 37°C. Verapamil was used as the positive inibitor of ABCB1. After washing three times with PBS, cells were analyzed with FCM as previously described [51].

### ABCB1 ATPase assay

The Vi-sensitive ATPase activity of ABCB1 in the membrane vesicles of High Five insect cells was measured as described in protocol. The membrane vesicles (100 μg of protein/ml) were incubated in ATPase assay buffer (50 mM MES, pH 6.8, 50 mM KCl, 5 mM sodium azide, 2 mM EGTA, 2 mM dithiothreitol, 1 mM ouabain, and 10 mM MgCl_2_) with or without 0.3 mM vanadate at 37°C for 5 minutes, then incubated with different concentrations of drugs at 37°C for 3 minutes. The ATPase reaction was incubated by the addition of 5 mM Mg-ATP. After incubating at 37°C for 20 minutes, the reactions were stopped by adding 0.1 ml of 5% SDS solution. The liberated inorganic phosphate (Pi) was measured as previously described [52].

### Docking protocol

The 3D structure of trametinib was obtained from the software ChemDraw 7.0. The refined crystal structure of mouse ABCB1 in complex with QZ59-RRR (PDB ID: 4M2S) and QZ59-SSS (PDB ID: 4M2T) [53] was obtained from the RCSB Protein Data Bank. Docking experiments were performed with Discovery Studio 3.0. The top-scoring pose ABCB1 complex was then subjected to energy minimization and used for graphical analysis.

### Statistical analysis

A student's t-test was used to compare individual data points among each group. A *P*-value of <0.05 was set as the criterion for statistical significance.

## SUPPLEMENTARY MATERIALS, FIGURES, TABLES


