# Supplementary material for: Trametinib modulates cancer multidrug resistance by targeting ABCB1 transporter
Source: Oncotarget. 2015 Apr 14;6(17):15494–509. doi: 10.18632/oncotarget.3820 (PMC4558166; doi:10.18632/oncotarget.3820)
Supplement: Supplementary file 1 [file oncotarget-06-15494-s001.pdf]

Trametinib modulates cancer multidrug resistance by targeting ABCB1 transporter

Supplementary Material

Supplementary Table 1: Summary of the potential residues in transmemberane (TM) domains of ABCB1 interacting with Trametinib in comparison with QZ59-RRR, QZ59-SSS and verapamil.

TM	Residue	QZ59-RRR	QZ59-SSS	Verapamil	Trametinib
**1**	His 60			x	
	Ala 63			x	
	Leu 64	x	x	x	
	Met 67		x		
	Met 68	x	x		x
	Phe 71	x	x		
**2**	Tyr114		x		
	Val121			x	
**4**	Ser218			x	
**5**	Met295		x		
	Phe299		x		x
	Ile302		x	x	x
	Tyr303		x		x
	Tyr306	x	x		x
**6**	Phe331				x
	Phe 332	x	x		x
	Leu 335		x	x	x
	Ile 336	x	x		x
	Ala 338			x	
	Phe 339	x	x		
**7**	Asn 717		x		x
	Gly718				x
	Gln 721	x	x		x
	Phe 724	x	x	x	x
	Ala725			x	x
	Phe 728	x	x		x
**8**	Phe 766		x		
**9**	Gln834				x
	Ala837			x	
	Asn838			x	x
**10**	Ile 864			x	
	Ala 867			x	
	Gly 868			x	
**11**	Phe938			x	
	Thr941			x	
	Met945		x		
	Tyr949	x	x		x
	Phe953		x		
**12**	Leu 971		x	x	
	Phe 974	x	x		
	Ser 975	x			x
	Val 978			x	

	Phe979	x	x		x
	Gly 980			x	
	Ala 981			x	
	Met 982	x	x		x
	Ala983	x	x		x
	Gln 986	x	x		x
	Val987		x		x
